# Sexual dysfunction among people with mental illness in Africa: A systematic review and meta-analysis study

**DOI:** 10.1371/journal.pone.0308272

**Published:** 2024-07-31

**Authors:** Setegn Fentahun, Mamaru Melkam, Gebresilassie Tadesse, Gidey Rtbey, Fantahun Andualem, Yilkal Abebaw Wassie, Gebremariam Wulie Geremew, Tekletsadik Tekleslassie Alemayehu, Tewodros Denekew Haile, Tilahun Nega Godana, Berihun Agegn Mengistie, Mulualem Kelebie, Girum Nakie, Techilo Tinsae, Girmaw Medfu Takelle

**Affiliations:** 1 Department of Psychiatry, College of Medicine and Health Science, University of Gondar, Gondar, Ethiopia; 2 Department of Medical Nursing, School of Nursing, College of Medicine and Health Sciences, University of Gondar, Gondar, Ethiopia; 3 Department of Clinical Pharmacy, School of Pharmacy, College of Medicine and Health Sciences, University of Gondar, Gondar, Ethiopia; 4 Department of Social and Administrative Pharmacy, School of Pharmacy, College of Medicine and Health Sciences, University of Gondar, Gondar, Ethiopia; 5 University of Gondar, College of Medicine and Health Science, School of Pharmacy, Department of Pharmaceutical Chemistry; 6 Department of Internal Medicine, School of Medicine, College of Medicine and Health Sciences, University of Gondar, Gondar Ethiopia; 7 Department of General Midwifery, School of Midwifery, College of Medicine and Health Sciences, University of Gondar, Gondar, Ethiopia; Taipei Veterans General Hospital, TAIWAN

## Abstract

**Background:**

Sexual dysfunction is the most frequent health problem among psychiatric patients. This could be the result of both the nature of the illness itself and the side effects of prescribed psychotropic medications. It also significantly affects an individual’s general well-being, interpersonal relationships, self-esteem, and treatment outcomes. Therefore, the current systematic review and meta-analysis was conducted to determine the combined prevalence of sexual dysfunction and its correlated factors among people with mental illness.

**Methods:**

We retrieved eligible primary studies using various search databases like PubMed, EMBASE, Science Direct, African Journal Online, Google Scholar, and Psychiatry Online. The report of this systematic review was reported following the Preferred Reporting Items for Systematic Review and Meta-Analysis (PRISMA) guidelines. We used standardized data extraction checklists and STATA version 14 for data extraction and analysis, respectively. The I-squared statistics test was used to check statistical heterogeneity within the included articles. Publication bias was assessed using a funnel plot and the Egger test. To estimate the overall prevalence and correlated factors of sexual dysfunction, a random effects model meta-analysis was employed.

**Results:**

In this meta-analysis, a total of 15 primary studies with 2849 psychiatric patients were included. The overall pooled prevalence of sexual dysfunction among psychiatric patients in Africa was 58.42% (95% CI: 49.55, 67.28). Having older age (OR = 1.92, 95% CI: 1.28, 2.87), longer duration of illness (OR = 2.60, 95% CI: 1.14, 5.93), history of relapse (OR = 3.51, 95% CI: 1.47, 8.43), poor quality of life (OR = 3.89, 95% CI: 2.15, 7.05), and antipsychotic medications (OR = 2.99, 95% CI: 1.84, 4.86) were significantly associated with sexual dysfunction.

**Conclusion:**

This meta-analysis revealed that approximately two-thirds of psychiatric patients in Africa are affected by sexual dysfunction. Therefore, the findings of this study recommend that when evaluating psychiatric patients, health professionals should focus more on sexual dysfunction. It is also essential to promote awareness and incorporate sexual health assessment and intervention into mental health services to reduce the overall burden of the problem.

## Introduction

Human sexual behavior is a complicated and natural aspect that can be affected by a wide range of physiological and psychological elements. The World Health Organization defines sexual health, as a condition of a person’s physical, emotional, mental, and social well-being about sexuality [1]. Respect and positivity towards sexuality and relationships are essential for maintaining good sexual health. It has been stated that a healthy sexual function is a basic component of quality of life and preserving a fulfilling intimacy [2]. Having enjoyable and secure sexual relationships free from compulsion, prejudice, and violence is another aspect of it. One of the most crucial features of sustaining a fulfilling personal relationship and a high quality of life is having healthy sexual functioning. Worldwide research on sexual attitudes and behaviors indicates that the most common sexual concern globally is the incapacity to have an orgasm during sexual activity and a lack of desire to initiate sex [3].

Sexual dysfunction can be described from a psychiatric aspect as a change in any one of the phases of the sexual response cycle, including sexual desire, excitation, orgasm, climax, resolution, and other issues such as dyspareunia and priapism [4]. The most frequent sexual dysfunction among women is reduced sexual desire, and men are most commonly affected by erectile dysfunction and premature ejaculation [5]. Psychiatric patients are more likely than the general population to experience sexual dysfunction, which may be linked to psychopathology, psychotropic medications, substance use, and psychosocial problems [6,7]. Interpersonal and sexual interactions are restricted by the nature of the mental illness itself. For instance, volition, anhedonia, and reduced feeling are common negative symptoms of schizophrenia that affect a person’s ability to engage in sexual activities, and sexual dysfunction is also a result of depressive symptoms like a lack of desire and energy [7,8]. Because of the stress reaction and hyperarousal state, anxiety plays a significant role in the occurrence of sexual dysfunction [9]. Numerous psychosocial problems are major contributing factors in the development of sexual dysfunction in psychiatric patients, such as poor interpersonal relationships (lack of communication, low-quality relationships, and unsolved disputes), low self-esteem, and a negative attitude towards sexual activity. Moreover, a history of sexual abuse or trauma has a significant and long-lasting impact on sexual dysfunction [10].

Sexual dysfunction can be caused by different psychiatric disorders, like mania, bipolar disorder, personality problems, especially borderline personality disorder [11,12]. In the USA, sexual dysfunction is estimated to affect 43% of women and 31% of men [13]. According to the findings of clinical and epidemiological studies, psychotropic medications, anxiety, and depression have a 30–70% effect on sexual dysfunction [7,14]. It has been found that 40%–65% of people with severe depressive illness have sexual dysfunction, and global sexual dysfunction was observed in about 35–45% of antidepressant-treated people [12,15]. Both male and female schizophrenic patients commonly experience sexual problems, which affects half of those patients on antipsychotic medications because of the nature of the disorder [16]. A meta-analysis study indicates that 30 to 60% of patients on Selective Serotonin Reuptake Inhibitors (SSRIs) may have sexual dysfunction caused by the medication’s side effects [17].

Sexual dysfunction is also associated with posttraumatic stress disorder, obsessive-compulsive and related disorders, and eating disorders because of the nature of these psychiatric disorders and their treatment modalities [7]. Substance use is another problem becoming more prevalent and contributing to sexual dysfunction. Prolonged use of alcohol causes erectile dysfunction, diminished sexual desire, and problems having an orgasm, and smoking may harm the nervous system, which is essential for the arousal of sexual desire. As a result, 26% of alcohol drinkers and 50% of smokers experienced sexual dysfunction [18,19].

There are various contributing factors to the development of sexual dysfunction among patients with psychiatric illness. Like long-term psychotropic medications, female sex, cigarette smoking, advancing age, illegal substance use, medication dosage, concurrent physical diseases, and the deterioration of interpersonal and sexual interactions as a result of the impacts of the illnesses [20–22]. On the other hand, sexual dysfunction contributes to marital stress, anxiety, despair, depression, poor self-confidence, low self-esteem, guilt, rage, and dissatisfaction in a relationship, which lowers the quality of life and leads to medication non-adherence [23,24]. It also has an effect on problems starting and maintaining a family and the development of suicidal behavior [25].

Although sexual dysfunction is more prevalent among patients with mental illness and influences the effects of psychotropic medications, it is neglected, frequently disregarded, and underdiagnosed, and most people with this problem fail to seek treatment. These could be because of feelings of shame and humiliation, fear of discrimination, or a marriage-threatening situation, and they do not believe the problem can be treated. Furthermore, most psychiatrists and other health professionals pay little attention to it and do not ask their clients about their sexual health during regular therapeutic sessions because of the discomfort of discussing sexual issues with clients. Several primary studies have been carried out in Africa to assess sexual dysfunction among psychiatric patients, but the results of these studies were extremely inconsistent, and there is a lack of representative data (combined prevalence) concerning sexual dysfunction in this population. Additionally, to the best of our knowledge, no previous systematic review and meta-analysis has been conducted on sexual dysfunction among psychiatric patients in Africa. Therefore, the current study aimed to estimate the pooled prevalence and correlated factors of sexual dysfunction among people with mental illness. The findings of the current study would be essential in filling an important research gap by determining the pooled prevalence of sexual dysfunction among African psychiatric patients in order to develop a common intervention strategy.

## Methods

### Data source and search strategy

The current systematic review and meta-analysis was conducted using both published and unpublished studies that examined the prevalence and associated factors of sexual dysfunction among psychiatric patients in Africa. The reports for this study were done based on the Preferred Reporting Items for Systematic Reviews and Meta-Analyses (PRISMA-2020) checklists [26] (S1 File). This review protocol was registered in the International Prospective Register of Systemic Review (PROSPERO) with registration number (ID = CRD42024535579). The primary articles were searched using PubMed/MEDLINE, African Journal Online, Psychiatry Online, EMBASE, Science Direct, and CINAHL. Additionally, Google and Google Scholar were also used to obtain articles. Moreover, to find additional papers, we looked through the retrieved studies’ reference lists. Articles were retrieved using the following search terms: “prevalence,” “magnitude,” "epidemiology,” “incidence,” “sexual dysfunction,” “associated factors,” “risk factors," "determinants," "predictors," "correlates," "mental illness," and “severe mental illness,” “psychiatric patients” and “Africa”. To combine the above mesh terms, we have used Boolean operators like “AND” and “OR”. The search of articles was performed independently by two authors (SF and MM) up to March 20, 2024 (S2 File).

### Selection and eligibility criteria

The following were the inclusion criteria for the present systematic review and meta-analysis: (1) all observational studies, like cross-sectional, case-control, and cohort study designs, that assessed the prevalence and associated factors of sexual dysfunction in psychiatric patients were eligible for this review; (2) primary articles with complete data and easily accessible; (3) studies written in the English language and conducted in Africa until 2024. Studies were excluded if (1) they did not report the outcome variables; (2) they had incomplete data and were not easily accessible; (3) case reports, reviews, case studies, conferences, and short communications; and (4) they were not conducted in Africa and published in English.

### Outcome measurements

The first outcome interest of this study was to evaluate the combined magnitude of sexual dysfunction among psychiatric patients. The second outcome of the current study was to determine the cumulative effects of variables that contribute to sexual dysfunction in African mentally ill patients. A two-by-two table was used to compute the odds ratio from the included primary articles.

### Data extraction and quality assessment

After retrieving the primary studies based on prespecified inclusion criteria, two authors (GT and FA) executed the data extraction using a standardized data extraction checklist constructed in Microsoft Excel. The final retrieved data from each included article contains the following study characteristics: corresponding author’s name, study design, the country where the study was conducted, publication year, sample size, sex, type of mental illness, prevalence of sexual dysfunction, screening tool, associated factors, 95% confidence interval, and odds ratio. Following data extraction, the third author (GMT) critically reviewed each table for the sake of uniformity. During the data extraction process, disputes and differences were settled by consensus and conversation. The Joanna Briggs Institute (JBI) critical evaluation checklist was used to evaluate the quality of the included primary articles [27]. The assessment tool was modified for use in observational studies, and a score of at least five out of nine was taken to be a good quality score. The quality of each included article was evaluated by two reviewers (GN and TT). Throughout the quality assessment, any differences between the two independent authors were resolved by contacting the third and fourth authors (GWG and TDH) (S3 File).

### Statistical analysis and synthesis

After the extraction of relevant data from each included primary article via Microsoft Excel format, the extracted data was exported to STATA version 14 statistical software for additional analysis. The results of the current study were presented and summarized using texts, tables, and forest plots with a 95% confidence interval. The presence of heterogeneity in the included studies was assessed using the inverse variance index (I^2^). The range of the I^2^ statistics test is 0 to 100%, and I^2^ values of 0, 25, 50, and 75% indicate no, low, moderate, and high heterogeneity, respectively [28]. Since we observed a high level of heterogeneity among the included studies, a random-effect meta-analysis model was employed to estimate the pooled prevalence. Subgroup analysis was done to identify the potential source of heterogeneity among primary articles. Moreover, to determine the effects of a single study on the combined prevalence, sensitivity analysis was employed. Visual inspection of funnel plots and Egger tests were used to assess the existence of publication bias [29,30]. An asymmetric distribution of the funnel plot confirmed the presence of publication bias, and according to Egger’s test, p-values < 0.05 have been taken as evidence of publication bias in the included studies.

## Results

### Selection and identification of studies

**Fig 1:**
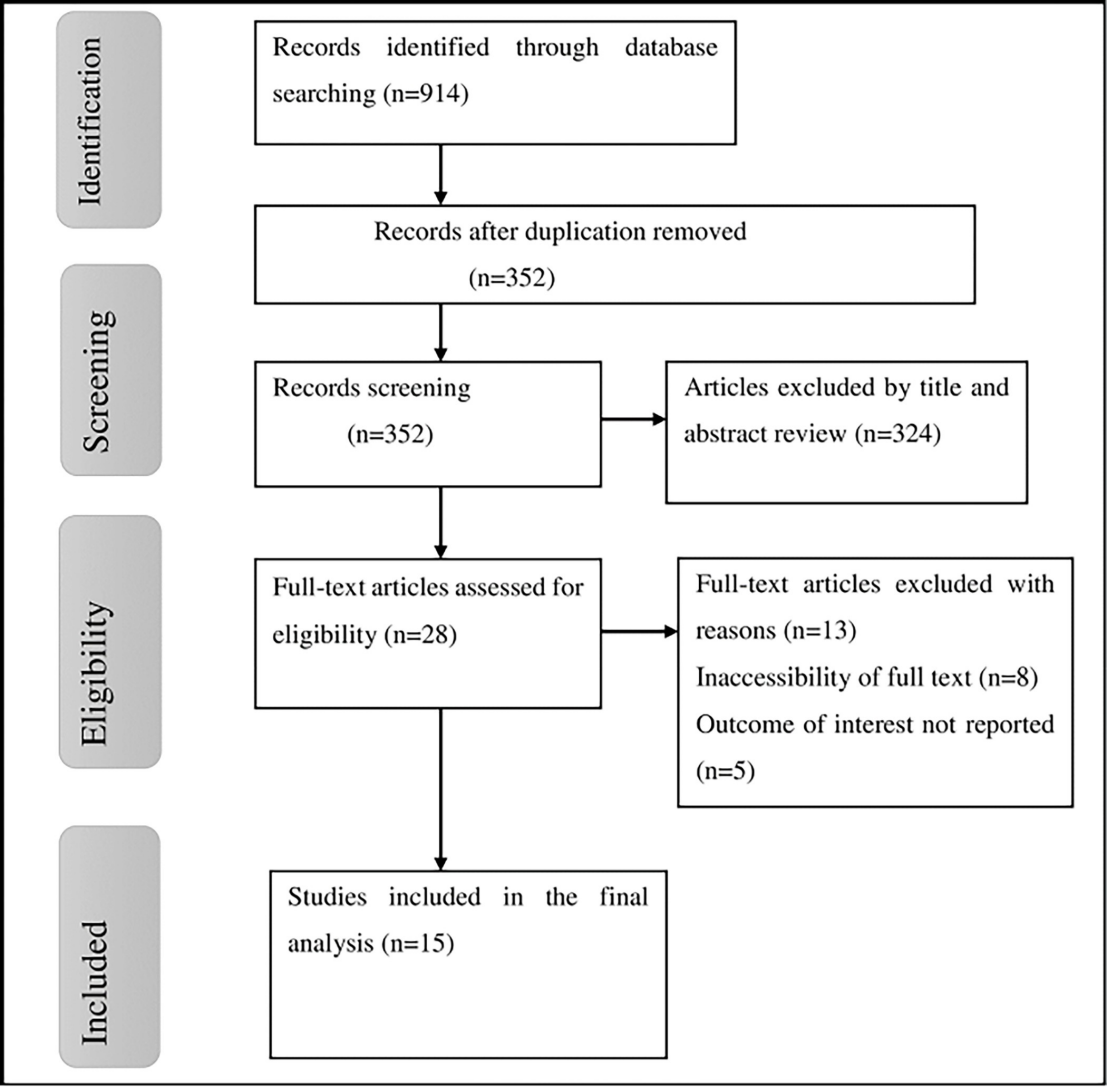
Flowchart of study selection for meta-analysis of sexual dysfunction aniong psychiatric patients in Africa.

A total of 914 studies were retrieved for the present systematic review and meta-analysis using various electronic databases. Among the retrieved articles, 562 duplicate records were removed. After reviewing the abstracts and titles, 324 papers were excluded because they did not meet our inclusion requirements, like not reporting outcomes of interest and studies not conducted in Africa. Moreover, the other 28 articles were also evaluated using prespecified inclusion criteria, and 13 studies were excluded for other reasons. Lastly, fifteen primary studies were included in the final analysis of this study (Fig 1).

### Description of included studies

In this study, a total of 2849 study participants were included. The current systematic review and meta-analysis contained a total of 15 full-text articles that examined sexual dysfunction and its contributing factors among psychiatric patients and were published until 2024. The included articles were conducted in the five African countries: six studies were conducted in Nigeria [31–36], four studies were carried out in Ethiopia [37–40], three articles were in Egypt [41–43], one study was done in South Africa [44] and the other one study was conducted in Tunisia [45]. Concerning the study design, most of the included studies (thirteen studies) were conducted using a cross-sectional study design, and the other two remaining articles were prospective cohort and case-control studies. The prevalence of included articles ranged from 35% [44] to 84.7% [31]. The included studies in this systematic review and meta-analysis were conducted using different assessment tools, such as CSFQ-14, IIEF, ASEX, and FSFI. Accordingly, six studies were assessed using CSFQ-14, five studies were done using IIEF, three studies used ASEX, and the remaining study was evaluated using FSFI. Out of the 15 included primary articles, nine studies were conducted among schizophrenic patients, and the other six studies were done among people with any mental illness (Table 1).

**Table 1:** Characteristics of studies included in this systematic review and meta-analysis on sexual dysfunction among psychiatric patients in Africa.

Authors	Publication year	Country	Study design	Tool	Type of illness	Sex	Sample size	Prevalence (%)
Fanta et al	2018	Ethiopia	C.S	CSFQ-14	schizophrenia	both	422	82.7
Tsehay et al	2020	Ethiopia	C.S	CSFQ-14	any mental illness	both	423	58.2
Sewalem et al	2022	Ethiopia	C.S	CSFQ-14	any mental illness	both	421	45.4
Ayalew et al	2024	Ethiopia	C.S	CSFQ-14	schizophrenia	both	419	66.3
Sabry et al	2017	Egypt	C.C	ASEX	schizophrenia	male	50	64
Abdelatti et al	2020	Egypt	C.S	IIEF	any mental illness	male	80	51.2
Saad et al	2015	Egypt	P.C	CSFQ-14	schizophrenia	both	80	61.25
Bram et al.	2014	Tunisia	C.S	CSFQ-14	schizophrenia	both	38	71
Adesola et al	2021	Nigeria	C.S	IIEF	any mental illness	male	144	84.7
Esan et al	2018	Nigeria	C.S	ASEX	schizophrenia	both	90	36.7
Osasona et al	2019	Nigeria	C.S	IIEF	any mental illness	male	150	48.7
Olisah et al	2016	Nigeria	C.S	FSFI	any mental illness	female	133	58.8
Oyekanmi et al	2012	Nigeria	C.S	IIEF	schizophrenia	male	279	40.4
Olose et al	2021	Nigeria	C.S	IIEF	schizophrenia	male	43	72.1
Luckhoff et al	2022	S.Africa	C.S	ASEX	schizophrenia	both	77	35

Note: C-S = Cross-sectional, C-C = Case-control, P.C = Prospective Cohort, S.Africa = South Africa.

### Prevalence of sexual dysfunction among psychiatric patients

**Fig 2:**
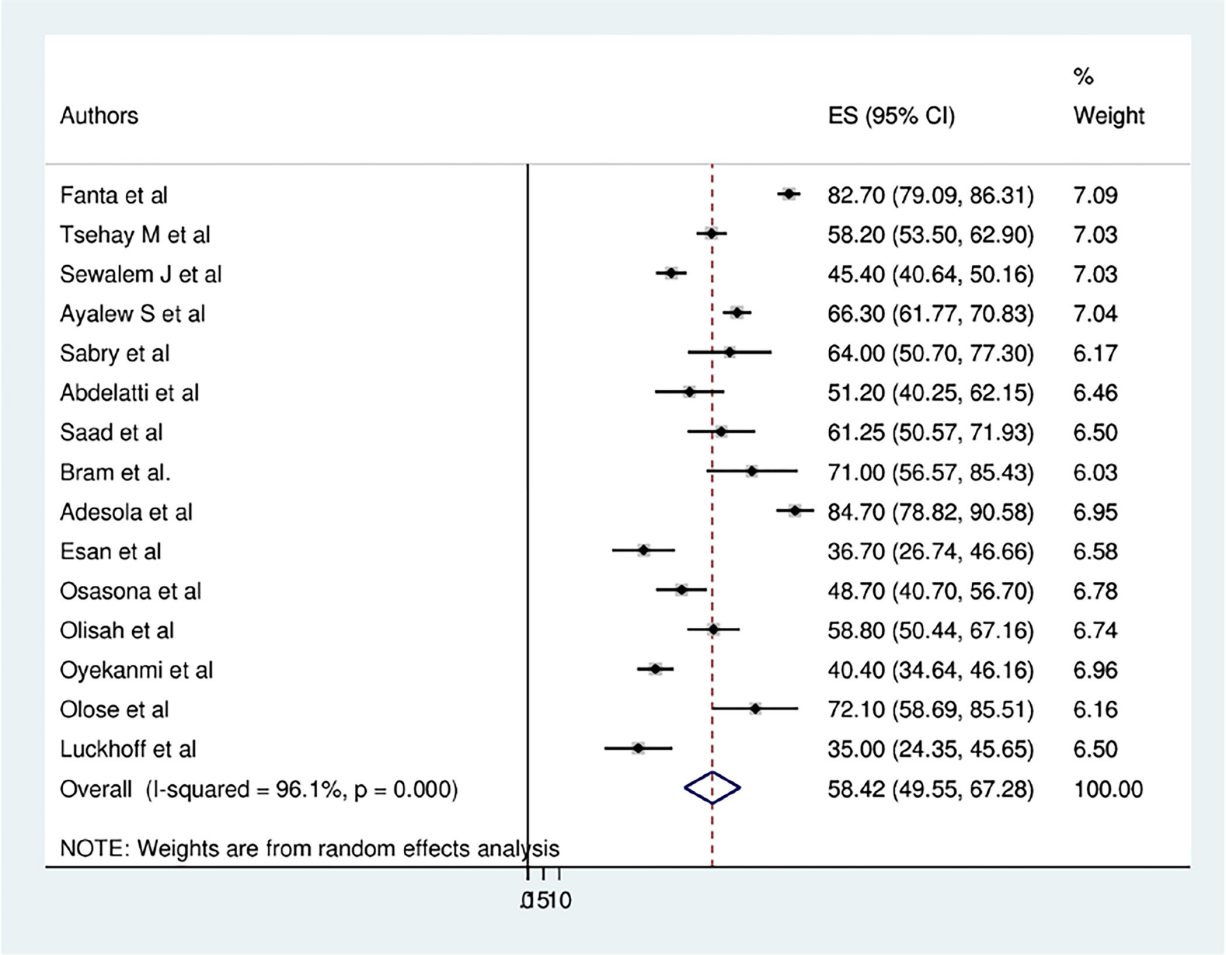
Forest plot of the pooled prevalence of sexual dysfunction among psychiatric patients in Africa.

The overall pooled prevalence of sexual dysfunction among psychiatric patients in Africa was found to be 58.42% (95% CI: 49.55, 67.28) (Fig 2). As shown from the result of the statistics test (I^2^), there was a high level of heterogeneity among the included primary studies (I^2^ = 96.1%, *P*≤0.001*)*. Therefore, to estimate the pooled prevalence of sexual dysfunction, a random-effects model was conducted. Moreover, a subgroup analysis was conducted using different study participants to identify the potential cause of the heterogeneity within the included articles.

### Subgroup analysis

Subgroup analysis was performed using the country where the study was conducted, screening tools, type of mental illness, sex, and study design. Accordingly, studies conducted in Ethiopia revealed the highest estimated prevalence of sexual dysfunction (63.20%; 95% CI: 47.04, 79.36). A higher prevalence of sexual dysfunction was observed among studies conducted in males (60.08%; 95% CI: 42.83, 77.32). The pooled prevalence of sexual dysfunction was 58.77% (95% CI: 45.68, 71.86) in studies done among schizophrenic patients, which was higher than in studies conducted on any type of mental illness (57.94%; 95% CI: 45.46, 70.43). The lowest prevalence of sexual dysfunction was found among studies that were conducted using a cross-sectional study design, and the highest was detected in studies conducted using other study designs (prospective cohort and case-control), with an estimated pooled prevalence of 57.81% (95% CI: 48.07, 67.55) and 62.33% (95% CI: 54.00, 70.65**)**, respectively. Furthermore, the results of subgroup analysis by assessment tools indicated a higher prevalence of sexual dysfunction among studies that were conducted using CSFQ-14 (64.02%; 95% CI: 51.21, 76.84) (Table 2).

**Table 2:** Subgroup analysis of sexual dysfunction among psychiatric patients in Africa.

Variables	Subgroup	Number of studies	Prevalence (95% CI)	I^2^ (%)	P value
Country	Ethiopia	4	63.20(47.04, 79.36)	98.2	0.000
	Nigeria	6	56.84(39.80, 73.88)	96.5	0.000
	Egypt	3	58.36(50.75, 65.98)	23.4	0.271
	Others*	2	52.66(17.38, 87.93)	93.5	0.000
Study design	Cross-sectional	13	57.81(48.07, 67.55)	96.7	0.000
	Others**	2	62.33(54.00, 70.65)	0.0	0.752
Sex	Both sex	8	57.18(44.98, 69.39)	96.9	0.000
	Male	6	60.08(42.83, 77.32)	96.0	0.000
	Female	1	58.80(50.44, 67.16)	-	-
Tool	CSFQ-14	6	64.02(51.21, 76.84)	97.0	0.000
	IIEF	5	59.34(39.58, 79.10)	96.2	0.000
	ASEX	3	44.72(28.05, 61.39)	84.9	0.001
	FSFI	1	58.80(50.44, 67.16)	-	-
Type of mental illness	Any mental illness	6	57.94(45.46, 70.43)	95.6	0.000
	Schizophrenia	9	58.77(45.68, 71.86)	96.4	0.000

*South Africa and Tunisia

**Case-control and Prospective Cohort.

### Meta-regression

We performed meta-regression in addition to the subgroup analysis in order to determine the source of heterogeneity. The study characteristics, like year of publication, sample size, and country were used in a meta-regression analysis. However, the findings of the meta-regression showed that there was no significant heterogeneity resulting from any of the previously mentioned study variables (Table 3).

**Table 3:** Meta-regression analysis of factors affecting study heterogeneity.

Variable	Coef.	Std. Err.	t	P>t	(95% Conf. Interval)
Year of publication	.0133459	.2607402	0.05	0.960	-.5499491 .5766408
Sample size	.0003979	.0059537	0.07	0.948	-.0124643 .0132601
Country	-.0959856	1.23946	-0.08	0.939	-2.77369 2.581718

### Publication bias and sensitivity analysis

**Fig 3:**
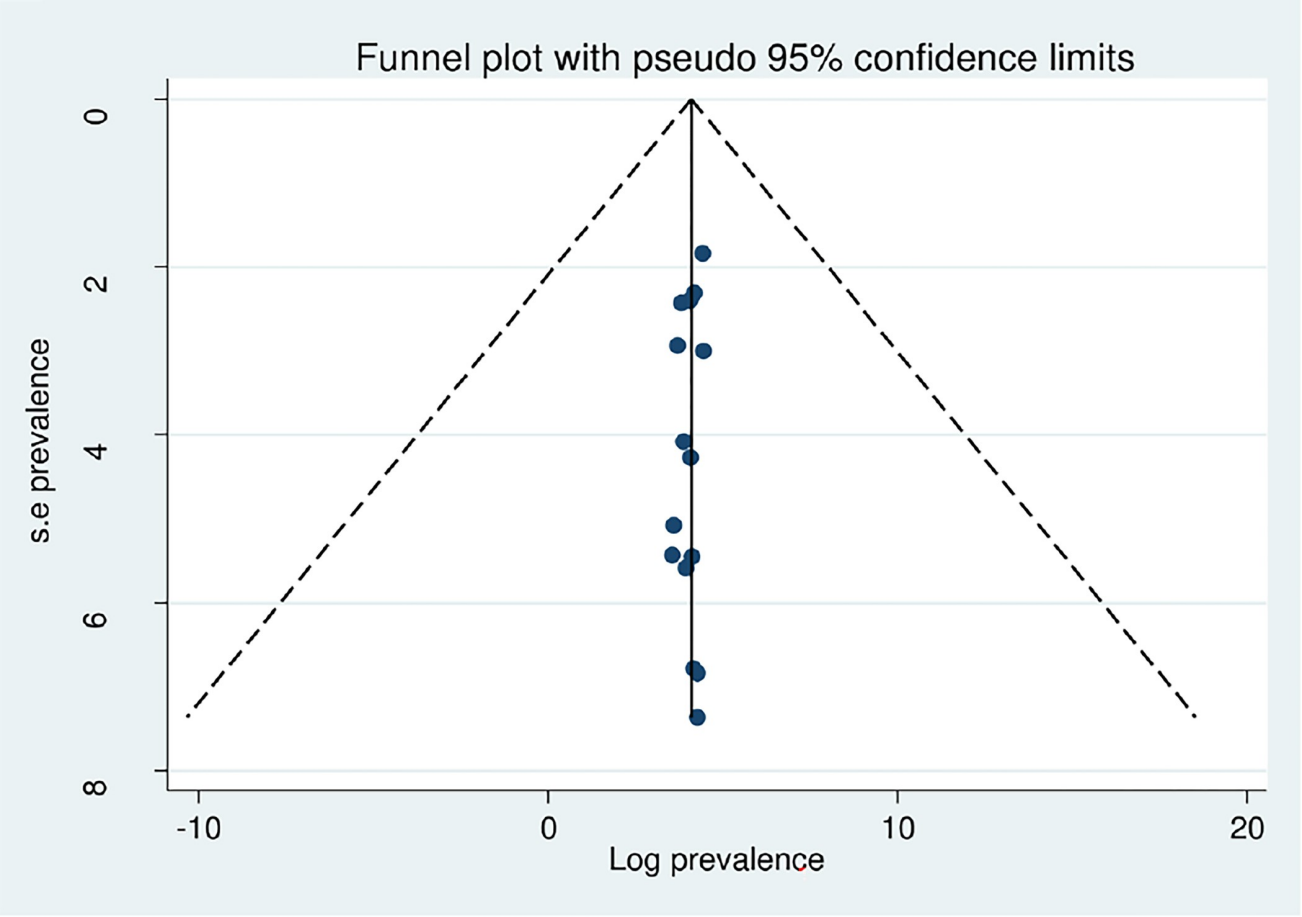
Funnel plot of included studies on sexual dysfunction among psychiatric patients.

Publication bias was checked by using both the funnel plot and the Eggers test. The symmetrical distribution of the funnel plot confirmed the absence of publication bias in the included articles (Fig 3). Additionally, the Eggers test findings showed that there was no publication bias in this systematic review and meta-analysis (P = 0.223) (Table 4). Concerning sensitivity analysis, due to the high level of heterogeneity in the included studies, we conducted a sensitivity analysis using a one-study leave-out technique to identify the effect of individual studies on the overall combined prevalence of sexual dysfunction. According to the results of the sensitivity analysis, the pooled prevalence of sexual dysfunction was not significantly impacted by excluding a single study from the analysis (Table 5).

**Table 4:** Eggers test of sexual dysfunction among psychiatric patients in Africa.

Std_Eff	Coef.	Std. Err.	t	P>t	(95% Conf. Interval)
Slope	.50268	10.31039	7.23	0.000	52.22842 .77693
Bias	.985865	3.113647	-1.28	0.223	-10.71249 2.740761

**Table 5:** Sensitivity analysis of sexual dysfunction among psychiatric patients in Africa.

Authors	Estimate 95% CI	Heterogeneity	
		I^2^	*P* value
Fanta et al	56.53(48.68, 64.37)	93.6	≤0.001
Tsehay et al	58.43(48.59, 68.27)	96.3	≤0.001
Sewalem et al	59.40(50.32, 68.49)	95.7	≤0.001
Ayalew et al	57.82(47.93, 67.72)	96.3	≤0.001
Sabry et al	58.05(48.80, 67.30)	96.4	≤0.001
Abdelatti et al	58.92(49.66, 68.17)	96.3	≤0.001
Saad et al	58.22(48.91, 67.53)	96.4	≤0.001
Bram et al.	57.61(48.40, 66.82)	94.4	≤0.001
Adesola et al	58.42(49.55, 67.28)	96.1	≤0.001
Esan et al	59.95(50.95, 68.94)	96.1	≤0.001
Osasona et al	59.12(49.84, 68.40)	96.2	≤0.001
Olisah et al	58.39(48.99, 67.79)	96.4	≤0.001
Oyekanmi et al	59.77(50.95, 68.59)	95.6	≤0.001
Olose et al	57.52(48.30, 66.74)	96.4	≤0.001
Luckhoff et al	60.04(51.07, 69.02)	96.1	≤0.001

### Factors associated with sexual dysfunction

**Fig 4:**
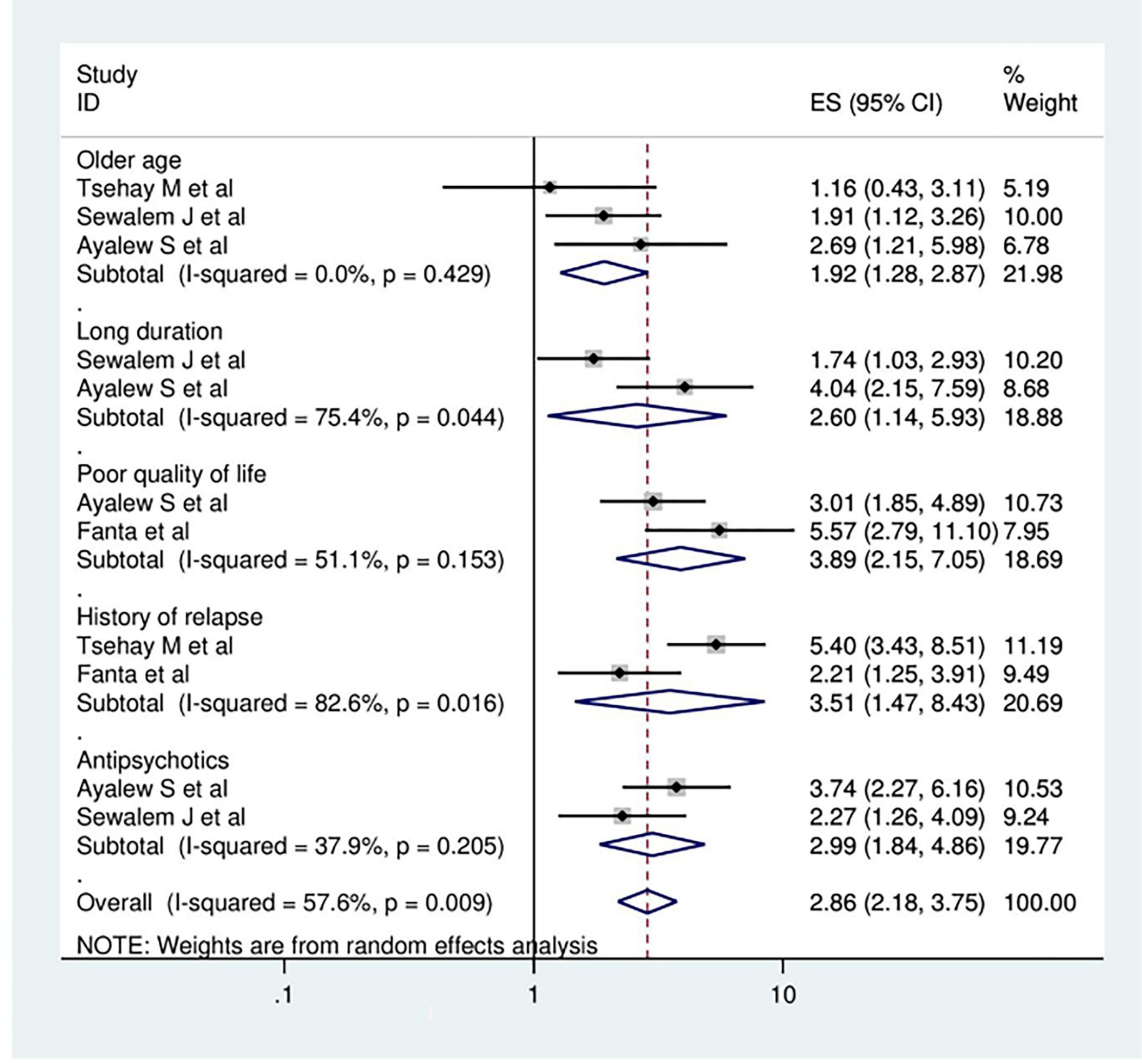
The Forest plot showing associated factors of sexual dysfunction among psychiatric patients in Africa.

In the current meta-analysis, numerous contributing factors have been identified as determinants of sexual dysfunction among psychiatric patients. The present systematic review and meta-analysis employed the associated factors that were mentioned in a minimum of two primary articles. The meta-analysis’s results showed that older age, long duration of illness, history of relapsing, and poor quality of life were found to be significantly associated with sexual dysfunction in mentally ill patients. Having older age was stated as a contributing factor to sexual dysfunction in the three primary articles. The odds of developing sexual dysfunction were 1.92 times higher among psychiatric patients who were older than their counterparts (OR = 1.92, 95% CI: 1.28, 2.87). According to the analysis of two studies, those respondents who had a longer duration of illness had 2.6 times greater odds of having sexual dysfunction compared to individuals with a shorter duration of the illness (OR = 2.60, 95% CI: 1.14, 5.93). The meta-analysis of two articles revealed that poor quality of life was significantly associated with sexual dysfunction among mentally ill patients. The combined odds ratio showed that study participants with a poor quality of life were 3.89 times more likely to develop sexual dysfunction as compared to participants with a good quality of life (OR = 3.89, 95% CI: 2.15, 7.05). In the present study, respondents who had a history of relapse were 3.5 times more exposed to sexual dysfunction than participants who had no history of relapse (OR = 3.51, 95% CI: 1.47, 8.43), and those study participants who were taking antipsychotic medications were 2.99 times more likely to develop sexual dysfunction compared to their counterparts (OR = 2.99, 95% CI: 1.84, 4.86) (Fig 4).

## Discussion

Sexual dysfunction is a complicated and multidimensional problem that has to be carefully considered and investigated in people with mental illness. In order to provide comprehensive care and enhance the quality of life, it is imperative to comprehend the prevalence, contributing factors, and consequences of sexual dysfunction among individuals with mental illness. The current systematic review and meta-analysis, to the best of our knowledge, is the first to offer information regarding the combined prevalence of sexual dysfunction and contributing factors among mentally ill patients in Africa. The results from the present study on the combined prevalence and correlated factors of sexual dysfunction may help psychiatric patients address the problem earlier. Furthermore, this review provides a valuable foundation for healthcare professionals, policymakers, and researchers on sexual dysfunction in people with mental illness, and it can also offer insightful information about the prevalence, contributing factors, and possible interventions for this frequently disregarded area of mental health.

The findings of the present systematic review and meta-analysis showed that the overall estimated prevalence of general sexual dysfunction among people with mental illness was found to be 58.42% (95% CI: 49.55, 67.28). which was in line with another study that was conducted among Chinese male schizophrenia patients with a prevalence of 60.7% [46].

The pooled prevalence of sexual dysfunction in the current review was higher than the studies conducted in different countries. In a systematic review and meta-analysis that was conducted in Chinese schizophrenic patients by including sixteen primary articles with 5417 study participants, the overall prevalence of sexual dysfunction was 50.43%, which is lower than the result of this systematic review and meta-analysis [47]. The possible reason for the observed inconsistency might be due to differences in sampled study participants. This study was conducted among individuals with all mental illnesses, but a study in China was carried out on only schizophrenic patients. The result of this meta-analysis was also higher than studies conducted in India (45%) [48] and the Netherlands (25%) [49]. This difference may be due to the fact that this study was conducted by including various primary articles to provide a pooled prevalence of sexual dysfunction, whereas a study carried out in India had a single finding. Socioeconomic disparities and variations in the availability of health services could be the other explanation for the observed discrepancy [50].

Conversely, the finding of this systematic review and meta-analysis was lower than those of studies conducted in India (70%) [51], China (69.9%) [46], and London (65%) [52]. The result of this meta-analysis was also lower than a pooled analysis of three vilazodone studies conducted in the United States of America (USA) among patients with generalized anxiety disorder, with a prevalence range of 65–94% of sexual dysfunction [53]. Differences in sample size, study participants, and sociocultural level could be the reason for the observed variations. The findings of subgroup analysis using country showed that the higher combined magnitude of sexual dysfunction was detected in Ethiopia (63.20%; 95% CI: 47.04, 79.36), and the lower estimated pooled prevalence was observed in other African countries (South Africa and Tunisia) (52.66%; 95% CI: 17.38, 87.93). This disparity may be related to variations in sample sizes, access to healthcare, and societal diversity among African countries.

Regarding contributing factors of sexual dysfunction, participants with old age had 1.92 times greater odds of developing sexual dysfunction compared to their counterparts. The finding was consistence with previous studies done in China [46] and USA [13]. A possible reason for this association could be that individuals of increasing ages may experience physiologic alterations such as lower testosterone levels in men and decreased estrogen release in women, which could impact sexual function and libido [54]. It is also related to physical illnesses associated with aging and psychological factors that contribute to sexual problems [55].

In this meta-analysis, those respondents who had a longer duration of illness were a contributing factor in the development of sexual dysfunction. This was supported by the reports of a previous study conducted in India [56]. This could be because when the disease worsens, the brain’s structure changes, biochemical processes are disrupted, and treatment resistance develops, which may impact sexual interactions [57].

In the present systematic review and meta-analysis, having a history of relapse was significantly associated with sexual dysfunction in individuals with mental illness. The possible explanation for the association is that sexual performance may be impacted by the illness’s worsening, which happens when relapses occur more frequently [58]. Those respondents with a poor quality of life were 3.89 times more likely to develop sexual dysfunction as compared to participants with a good quality of life. This result was in line with other studies carried out in Turkey [59] and New York [8]. This could be due to the fact that one of the main determinants of quality of life is having a satisfying personal connection, which can be impacted by poor sexual functioning [60]. Concerning psychotropic medications, those respondents who were taking antipsychotic medications had 2.99 times greater odds of having sexual dysfunction compared to those who were not taking antipsychotic medications. The possible reason might be that since dopamine is essential for controlling arousal and sexual desire, the blocking of dopamine can result in sexual dysfunction [61].

### Strengths and limitations of the study

Even though the present meta-analysis and systematic review employed extensive search methods to generate a combined estimated prevalence and related factors of sexual dysfunction, there are various limitations to this study. The first limitation was that this study was conducted in some African countries, and most of the primary articles included in this systematic review had a small sample size, which may affect the representativeness. Second, this systematic review and meta-analysis was conducted by including most of the cross-sectional studies. Finally, a high level of heterogeneity was observed in this study.

## Conclusion

The current systematic review and meta-analysis revealed that the pooled prevalence of general sexual dysfunction among individuals with mental illness in Africa was high. The prevalence of sexual dysfunction differs across the included countries; the highest prevalence was observed in Ethiopia. This study indicated that older age, long duration of illness, history of relapse, poor quality of life, and antipsychotic medications were positively associated with sexual dysfunction. Therefore, the findings of this study recommend that sexual dysfunction requires a comprehensive strategy such as psychosocial interventions, patient education, pharmaceutical management, and routine evaluations that consider contributing factors. It is also important for mental health professionals to be aware of sexual dysfunction when assessing and managing individuals with mental illness by asking patients about this issue gently and sensitively. Moreover, clinicians need to be more informed of the sexual side effects of psychotropic medications to alleviate the burden of sexual dysfunction in this vulnerable population. Lastly, interventional studies should be part of future studies to further understand the cause and long-term effects of sexual dysfunction in psychiatric patients.

## Supporting information

S1 File PRISMA checklist for sexual dysfunction. (DOCX)

S2 File Searching strategy. (DOCX)

S3 File Quality assessment. (DOCX)

## Abbreviations

ASEX: Arizona Sexual Experience Scale
CI: Confidence Interval
CSFQ: Changes in Sexual; Functioning Questionnaires
FSFI: Female Sexual Function Index
IIEF: International Index of Erectile Function
JBI: Joanna Briggs Institute
OR: Odd Ratio
PRISMA: preferred Reporting Items for Systematic Reviews and Meta-Analysis
USA: United States of America
