# Supplementary material for: Sexual dysfunction among people with mental illness in Africa: A systematic review and meta-analysis study
Source: PLoS One. 2024 Jul 31;19(7):e0308272. doi: 10.1371/journal.pone.0308272 (PMC11290669; doi:10.1371/journal.pone.0308272)
Supplement: S2 File — (DOCX) [file pone.0308272.s002.docx]

**Additional file 2: Search strategy**

**PubMed**

(“Sexual dysfunction” [All Fields] AND “Prevalence” [All Fields] OR “Magnitude” [All Fields] OR “Epidemiology” [All Fields] OR “Incidence” [All Fields] AND “Associated factors” [All Fields] OR “Risk factors” [All Fields] OR “Determinants” [All Fields] OR “Predictors” [All Fields] OR “Correlates” [All Fields] AND “Mental illness” [All Fields] OR “Severe mental illness” [All Fields] OR “Psychiatric patients” [All Fields] AND “Africa” [All Fields]).

**EMBASE and CINAHL**

“Sexual dysfunction AND Prevalence OR Magnitude AND Associated factors OR Determinants AND mental illness OR psychiatric patients AND Africa”.

**Google Scholar and African Journal Online**

Sexual dysfunction AND Prevalence OR Magnitude AND Associated factors OR Determinants AND severe mental illness OR psychiatric patients OR mental illness AND Africa.

**Science Direct**

Sexual dysfunction AND Prevalence AND Associated factors AND mental illness AND Africa.
