# Supplementary material for: Sexual dysfunction among people with mental illness in Africa: A systematic review and meta-analysis study
Source: PLoS One. 2024 Jul 31;19(7):e0308272. doi: 10.1371/journal.pone.0308272 (PMC11290669; doi:10.1371/journal.pone.0308272)
Supplement: S3 File — (DOCX) [file pone.0308272.s003.docx]

Supplemental file 2: Quality assessment of sexual dysfunction and its associated factors among psychiatric patents in Africa included studies in this review.

| Authors | Q1 | Q2 | Q3 | Q4 | Q5 | Q6 | Q7 | Q8 | Q9 | Total score (9%) |
| --- | --- | --- | --- | --- | --- | --- | --- | --- | --- | --- |
| Fanta et al | Y | Y | Y | NA | Y | Y | Y | Y | Y | 8 |
| Tsehay et al | Y | Y | Y | Y | Y | Y | Y | Y | Y | 9 |
| Sewalem et al | Y | Y | Y | Y | Y | Y | Y | Y | Y | 9 |
| Ayalew et al | Y | Y | Y | NA | NA | Y | Y | Y | Y | 7 |
| Sabry et al | Y | Y | NA | Y | Y | Y | Y | Y | Y | 8 |
| Abdelatti et al | Y | NA | NA | Y | Y | Y | Y | Y | Y | 7 |
| Saad et al | Y | Y | NA | Y | Y | Y | Y | Y | Y | 8 |
| Bram et al. | Y | Y | NA | Y | Y | Y | NA | Y | Y | 7 |
| Adesola et al | Y | Y | Y | Y | Y | Y | Y | Y | Y | 9 |
| Esan et al | Y | Y | Y | NA | Y | Y | Y | Y | Y | 8 |
| Osasona et al | Y | Y | Y | NA | Y | Y | Y | Y | Y | 8 |
| Olisah et al | Y | Y | Y | Y | Y | Y | Y | Y | Y | 9 |
| Oyekanmi et al | Y | Y | Y | Y | Y | Y | Y | Y | Y | 9 |
| Olose et al | Y | NA | Y | Y | NA | Y | Y | Y | Y | 7 |
| Luckhoff et al | Y | Y | NA | Y | Y | Y | Y | Y | Y | 8 |

**Key:** **Y**= Yes; **NR**= Not reported, **NA**=Not appropriate

**Question codes:**

1. Was the sample frame appropriate to address the target population?

2. Were study participants sampled in an appropriate way?

3. Was the sample size adequate?

4. Were the study subjects and the setting described in detail?

5. Was the data analysis conducted with sufficient coverage of the identified sample?

6. Were valid methods used for the identification of the condition?

7. Was the condition measured in a standard, reliable way for all participants?

8. Was there appropriate statistical analysis?

9. was the response rate adequate, and if not, was the low response rate managed appropriately?
